# Simulation of Clinical Visits as a Novel Approach to Evaluate Digital Health in Multiple Sclerosis: Simulation Study

**DOI:** 10.2196/67845

**Published:** 2025-12-11

**Authors:** Riley Bove, Luca Capezzuto, Imogen West, Simon Dryden, Saira Ghafur, Jack Halligan, Stanislas Hubeaux, Agne Kazlauskaite

**Affiliations:** 1 Department of Neurology, UCSF Weill Institute for Neurosciences, University of California, San Francisco, San Francisco, CA, United States; 2 F. Hoffmann-La Roche Ltd, Basel, Switzerland; 3 Roche Products Limited, London, United Kingdom; 4 Prova Health, Bermondsey Street, London, SE1 3XF, United Kingdom, +44 7742161980

**Keywords:** clinical simulation, digital health, digital health technology, multiple sclerosis, smartphone, teleconsultations, patient remote assessment, impact on clinical workflows, clinical utility

## Abstract

**Background:**

Efforts are being made to integrate digital health technologies into clinical care for multiple sclerosis (MS) to improve patient monitoring. Efficiently probing how they might impact clinical care could streamline digital tool development. The Floodlight digital tool, comprising 5 smartphone sensor–based tests, was used to generate health-related data on patient function and symptoms in a clinical simulation.

**Objective:**

The study had 3 objectives: (1) assess the utility of simulated clinical encounters as a research methodology for exploring the introduction of digital health technologies into clinical practice in MS, (2) confirm the fidelity of the simulated environment and patient cases developed and understand what metrics (eg, workflow, comprehensive evaluation) could be generated, and (3) generate insights into the utility of digitally collected data, including usability, clinical decision contribution, and impact on workflows, in clinical practice.

**Methods:**

A total of 2 patient cases consisting of clinical, radiological, and digital health data were developed with clinician input. US-based neurologists prepared for and conducted 2 simulated teleconsultations each, with an actor briefed on case profiles. Floodlight data were available, via the Floodlight MS™ Health Care Professional Portal, for 1 of the 2 consultations. Participant neurologists completed interviews and surveys assessing the fidelity of the cases presented, user experience and workflow metrics, patient concerns identified, care decisions made, and confidence in making decisions.

**Results:**

All 10 neurologists indicated that the simulations were high-fidelity representations of real consultations. Using the Floodlight technology for the first time, median time taken to prepare for and conduct the consultation was ~1.7‐2 minutes longer, with slightly greater mental effort reported by participants, compared with not using the tool. The Floodlight MS Health Care Professional Portal scored an “above average” 79 on the System Usability Scale and an “acceptable” Net Promoter Score of 10. In total, 6 of the 10 neurologists “strongly agreed” that it was easier and quicker to identify patient concerns when they had access to the patient-generated Floodlight data to prepare for their encounters than when they did not. Overall, more care and management decisions were taken when the digital tool was used (37 vs 29). Of those 37 decisions, Floodlight data were reported as a trigger for 20 decisions, always in combination with other elements including patient history (20/20) and clinical exam findings (9/20).

**Conclusions:**

These findings advance our understanding of clinical simulation as a method for evaluating digital tools and other innovative technologies for MS care. High-fidelity patient cases could be provided for the mock teleconsultations, and the simulated clinical environment was useful for evaluating usability and utility of a new digital tool—yielding preliminary evidence on how digital data could be accessed and utilized by neurologists to support routine MS care.

## Introduction

### Background

Multiple sclerosis (MS) is a central nervous system disorder marked by significant heterogeneity in its clinical presentation and characterized by disease progression from onset [1,2]. MS, often commencing with subtle signs and advancing to substantial disability, manifests through a wide spectrum of symptoms, ranging from sensory disturbances and motor deficits to cognitive impairments and emotional challenges, profoundly impacting patients, their families, and society [3,4].

To date, monitoring in MS has been reliant upon brief, infrequent clinical assessments (typically occurring once or twice annually, or at the time of a relapse), without sufficiently sensitive and objective (ie, free from rater-dependent variability) assessments of progression available to health care professionals (HCPs) [5]. Moreover, due to time constraints, standard assessments of disability in MS, such as the Expanded Disability Status Scale (EDSS) [6], which requires ~30 minutes to conduct [7], are not always performed in clinical appointments. Remote consultations (teleconsultations) are a recognized alternative to clinic visits [8] and have been adopted by the MS community, particularly during the COVID-19 pandemic [9,10].

Digital health technologies offer greater access to care in MS and opportunities to improve frequent monitoring [11], enabling the capture of ongoing subtle or subclinical progression of MS [1,12-14] in specific domains such as cognition (which can often be missed). These technologies can be remotely administered and are patient-centered and operated, allowing longitudinal assessment of multiple functional domains in a naturalistic environment [15,16]. Furthermore, they offer the opportunity to improve the involvement of patients in their disease or treatment course [17,18] and to enhance conversations between HCPs and people with MS [19]. By providing objective data and bridging the gap of information in between clinical visits, sensor-based digital assessments may ultimately allow HCPs to make more informed decisions, evaluate treatment effects, and act earlier on indications of disease progression [11] or symptomatic exacerbation prior to scheduled clinical visits.

Despite the potential benefits, evaluating, testing, and optimizing the integration of new and innovative solutions into health care settings can itself be a time- and resource-consuming process. Hence, there is growing demand for agile methods to rapidly learn how digital solutions can play a role in clinical practice [20]. Clinical simulation, a low-cost and scalable methodology whereby data are collected from users as they perform tasks in realistic clinical scenarios, can rapidly generate new insights. For example, they allow the exploration of the possible impact of novel methodologies without the risks inherent in deploying them in real clinical settings (eg, of misdiagnoses [21,22]) or of the expansion of technology to new user groups or in new clinical contexts [22]. The preliminary insights generated from simulation models can support the refinement of technologies before rollout or scaling up [23]. To confirm that the insights gained from these clinical simulations are meaningful, they must be of high fidelity and account for a number of variables (including behavioral ones) and the differences inherent among real-world practices [21,22].

### Aims and Objectives

This feasibility study was designed to evaluate clinical simulation as a method for assessing the preparation and conduct of teleconsultations. This included evaluating the fidelity of the simulated environment (compared with real practice) and the patient cases developed. An additional aim was to assess the feasibility of using data derived from smartphone-based remote assessment technology in combination with MS teleconsultations, including their usability and impacts on clinical workflows (eg, preparation time and time to identifying primary patient concerns), care decisions, and disease management. The Floodlight technology [24] was utilized as the digital tool for evaluation. Accordingly, the aim of this study was to evaluate the potential impact of integrating Floodlight MS into clinical practice, generating insights that may also be relevant to similar digital health tools. As it is expected that any new test or technology will initially impose a higher mental load on clinicians, the study also aimed to quantify the extent of this increase.

Floodlight MS comprised 5 smartphone sensor–based tests (hereafter referred to as “smartphone-based tests”) presented on an app, measuring information processing speed (Cognitive Test [25]), hand function (Pinching Test [24] and Draw a Shape Test [26]), and gait (U-Turn Test [27] and 2-Minute Walk Test [28,29]), as well as a Patient Journal including 2 patient-reported outcome (PRO) measures (Daily Status Questions and a Symptom Tracker [30]). The data were consolidated in a dashboard for HCPs (Floodlight MS HCP Portal), which was accessible between clinical visits [5], and constituted the focus of the technological assessment in this research.

## Methods

### Recruitment of Neurologists

US-based neurologists were recruited from Prova Health’s existing network, professional communities (eg, LinkedIn), and academic publications, and through snowball sampling (where research participants recruit future participants), and they were sent email invitations to take part in the study. Neurologists were required to have managed a minimum of 40 people with MS per month and to have had no previous experience using the Floodlight technology. Convenience sampling was utilized to recruit primarily academic neurologists, as they were expected to give more granular feedback on the technology. There was no defined sample size due to the exploratory nature of this feasibility study.

### Patient Case Overview

A total of 2 patient cases were presented to each participant neurologist. Radiology reports and clinical letters were drafted by qualified radiologists and medical doctors to describe the cases and reflect the real magnetic resonance imaging and clinical data in a realistic narrative. For additional authenticity, synthetic social and family histories consistent with typical MS presentations were included. All cases were validated by 2 expert neurologists.

The goal was to achieve high-fidelity patient cases that were representative of real-world cases while minimizing the variation inherent among people with MS. Patient cases included (experimental) or did not include (control) Floodlight data, thus simulating real-world scenarios in which neurologists manage people with MS with or without access to the Floodlight technology (see the *Patient Case Development* section for more details).

### Patient Actor

A professional actor was recruited to play the patients’ roles during the teleconsultations. They were recruited from a company with 25 years of experience in providing trained actors to simulate patients with medical, surgical, and psychiatric conditions. The patient roles were based on generated behavioral profiles, which were used to inform the biographies for the 2 patient cases. The actor was provided with detailed scripts developed by a medical doctor and validated by neurologists to ensure [1] the consistency and correctness of answers.

### Conduct of Clinical Simulations

Each clinical simulation session lasted approximately 2.5 hours and was conducted via Zoom. Medically qualified team members from Prova Health served as moderators, facilitating introductions (~5 min), reviewing the simulation session guide with the neurologists (~5 min), and providing training on the Floodlight technology (~30 min). After the simulated teleconsultations had begun, the moderators took notes with their cameras and microphones switched off. Teleconsultations were designed to replicate those that the participant neurologists would perform in their clinical practices. The individual simulated teleconsultation sessions for each of the 2 presented patient cases consisted of preparation (~5 min) and conduct (~30 min) phases, both led by the participant neurologist. Participant neurologists were instructed to evaluate the relevant information and recommend an appropriate management plan. In the preparation phase, clinical information provided with or without Floodlight data was reviewed for each patient case. The “patient” (played by the professional actor) then joined the call. Following the conclusion of each simulated teleconsultation, participant neurologists completed a short survey (~5 min) to collect feedback on the examined case. At the end of both sessions, the moderator shared a link to the postsimulation survey (~7 min) and conducted a brief interview (~5 min) with each participant neurologist.

### Patient Case Development

The 2 patient cases presented to each participant neurologist were developed using deidentified clinical and Floodlight data (see the *Patient Cases Selection Criteria* section). For both cases, the participant neurologists reviewed data from 3 recent clinical visits, including patient demographics, symptoms, diagnosis, treatment and medical history, and radiology reports based on original magnetic resonance imaging metrics.

The clinical data presented in the letters included EDSS scores, Symbol Digit Modalities Test (SDMT) scores, 9-Hole Peg Test times, and Timed 25-Foot Walk times. The main clinical traits emerging from clinical case narratives are presented in Table 1. It should be noted that although both patient cases presented gait impairment, Patient Case B was designed to show significant worsening of gait impairment in the last year.

**Figure 1.**
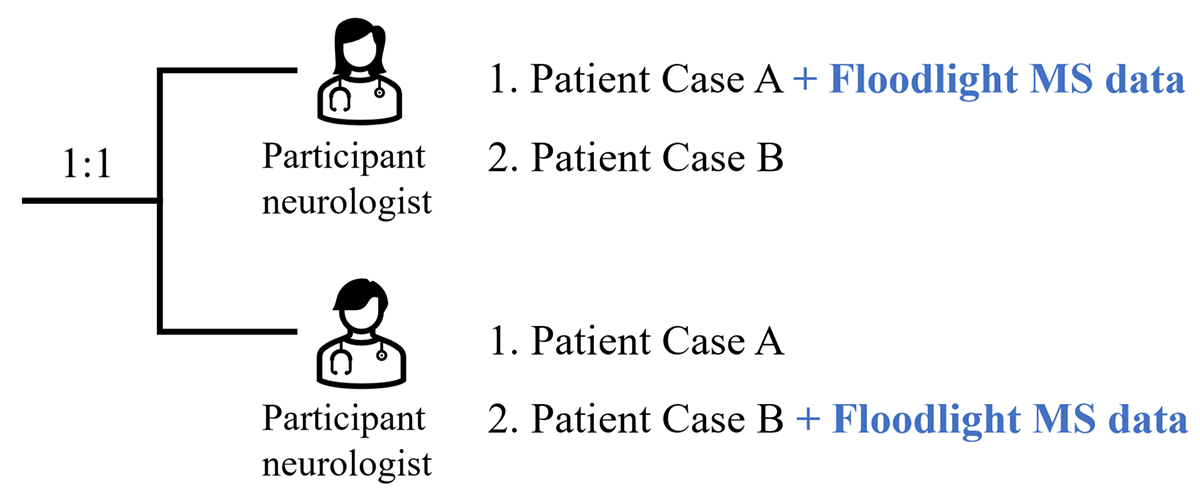
Randomization of patient cases before the initiation of teleconsultation. The order of cases and experimental conditions was randomized between participant neurologists, and consultations were conducted with 2 patient cases (A and B). Half (n=5) of the participant neurologists were randomized to have access to the Floodlight MS HCP Portal for the first case, while the other half had access to the solution for the second case. Due to a randomization error, 6 out of the 10 participant neurologists were assigned to the Floodlight MS HCP Portal for Patient Case A, instead of 5 out of 10. HCP: health care professional; MS: multiple sclerosis.

Floodlight data visualized in the Floodlight MS HCP Portal were presented for 1 of the 2 cases reviewed by each neurologist. The orders of the patient cases and of the interventions were randomized before the participant neurologists started the teleconsultations (Figure 1).

**Table 1.** Clinical traits, per patient case, presented during teleconsultation simulation.

Simulation patient case	Main clinical traits emerging from clinical case narratives and actor’s interpretation
Patient Case A	Cognitive function decline: decrease in SDMT^a^ score of ≥20% in the previous 12 months Hand motor function decline: increase in 9HPT^b^ time of ≥20% in the previous 6‐12 months History of gait impairment, as per T25FW^c^ test data Bowel and bladder issues reported in the previous 12 months Vision changes in previous history Fatigue
Patient Case B	Gait capacity decline: increase in T25FW time of ≥20% in the previous 6‐12 months Reported bladder issues in the previous 12 months Reported mood issues in the previous 12 months Spasticity and pain in the previous 12 months

a SDMT: Symbol Digit Modalities Test.

b 9HPT: 9-Hole Peg Test.

c T25FW: Timed 25-Foot Walk.

### Data Sources

Clinical data from the CONSONANCE study (NCT03523858) were used in conjunction with Floodlight performance and symptom tracker data collected over 12 consecutive months. CONSONANCE is an ongoing, phase 3b, 4-year, open-label study assessing the effectiveness and safety of ocrelizumab in adults with progressive multiple sclerosis.

Participants enrolled in the CONSONANCE study performed smartphone-based tests using a precursor to Floodlight MS for clinical research purposes, providing a battery of digital assessments and digital PROs. Data from the Pinching Test, the Draw a Shape Test, the U-Turn Test, and the 2-Minute Walk Test (all performed daily) were utilized, alongside data from a version of the Cognitive Test (performed weekly; involving matching a sequence of symbols to digits within 90 s).

Data from 2 PRO measures collected via the Floodlight Patient Journal were also used. These were Daily Status Questions (“How are you feeling physically now?” and “How is your mood now?”) and a biweekly Symptom Tracker to track symptoms related to cognition (attention deficit, memory loss, brain “fog” sensation, mental fatigue), upper extremity function (sensory disturbance, muscle weakness, clumsiness, muscular spasm, pain), lower extremity function (sensory disturbance, muscle weakness, clumsiness, muscular spasm, pain), gait (trouble walking, lack of balance, walking fatigue), and bowel or bladder dysfunction (incontinence, bladder or urinary problems, constipation), as well as the time that any new or worsening symptoms manifested and whether or not this was perceived to be a relapse. All Floodlight data collected in the CONSONANCE trial and used in this study were imported into the Floodlight MS HCP Portal (version 1.5 at the time of the research).

### Patient Cases Selection Criteria

Patient profiles were selected from the CONSONANCE dataset based on selected criteria that indicated a clinical performance decline observed in the previous 6‐12 months, based on standard clinical assessments of cognitive function, hand motor function, or gait capacity.

For Patient Case A, the key selection criteria included cognitive function decline (a decrease in SDMT score of ≥20% in the previous 12 mo) and hand motor function decline (increase in 9-Hole Peg Test time of ≥20% in the previous 6‐12 mo). For Patient Case B, the criteria included gait capacity decline (increase in Timed 25-Foot Walk time of ≥20% in the previous 6‐12 mo) as well as 2 consecutive reports of fatigue and bladder issues (score of 3‐6; some of the most impactful “invisible” symptoms of MS sourced via the SymptoMScreen). Some of the symptoms experienced by people living with MS may not be externally evident or easy to report and quantify, such as fatigue, mood disorders, cognitive impairments, pain, bladder or bowel dysfunction, sexual dysfunction, and vision changes. These symptoms can be difficult for the patient to report and articulate during a clinical encounter (eg, due to subjectivity bias, poor recall, or stigma), and for physicians to capture (limitations in available measurement tools, ample reliance on patient recall on the visit day) [31]. The clinical performance decline, together with reported signs of “invisible” symptoms, provided impetus for a care decision to be taken during the clinical simulation.

### Data Extraction

**Figure 2.**
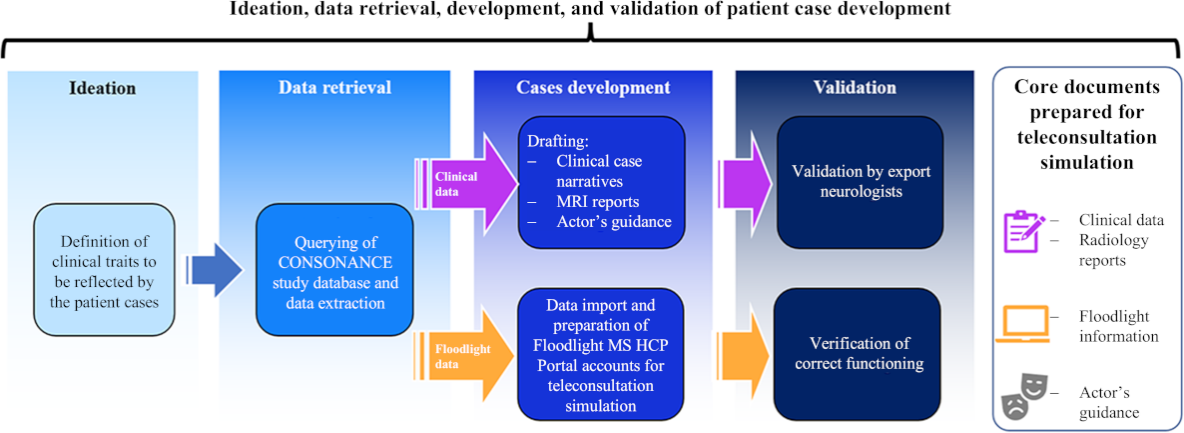
Clinical data extraction for teleconsultation simulation. Clinical data on ideation, data retrieval, case development, and validation of patient cases were extracted for teleconsultation simulation. Parameters such as multiple sclerosis (MS) phenotype, clinical factors, symptom reports, and synthetic data such as age and sex were included to provide transparency in demographic data. Floodlight data from the most recent 12-month period for each clinical profile were extracted for one of the cases presented to each participant neurologist. HCP: health care professional; MRI: magnetic resonance imaging.

Clinical data from 3 recent in-person clinical visits were extracted to present to the participant neurologists (Figure 2). No clinical data from the final in-person visit (the time point corresponding to the day of the teleconsultation in the simulation) were provided. Of note, cognitive decline had been confirmed at this visit for the patient in Patient Case A (as measured by the SDMT); these data were not provided to the participant neurologists in order to mimic a common clinical scenario in MS, where evident patient deterioration may precede formal confirmation through standard clinical assessments.

To build the patient cases, MS phenotype and date of diagnosis; clinical parameters, including recent disease history (eg, relapses, EDSS evolution); symptom reports; and current and prior treatments (eg, disease-modifying therapies) were also extracted. Additional “synthetic” data were used to construct demographic (age, sex) and radiology reports.

Floodlight data from the prior 12-month period for each clinical profile were also extracted to be provided for 1 of the 2 cases presented to each participant neurologist.

### Study Outcomes and Data Collection

Study outcomes were either moderator-reported or participant neurologist–reported (via participant surveys and interviews; Table 2). Measures included questions regarding the fidelity of the simulated cases developed; use of the digital tool (Floodlight MS), including user experience metrics for data visualization, overall experience (Net Promoter Score), and usability (System Usability Scale [32]); and workflow metrics, including time for case preparation, teleconsultation time, and mental effort required (Paas scale [33]). Confidence in approaching clinical consultations was also measured. Additionally, moderators recorded the time taken for the identification of patient concerns, the recognition of “invisible” MS symptoms, and the care decisions prompted and taken (including change in treatment management) with versus without having Floodlight data available.

A medically qualified moderator from the research team observed each simulation session and recorded outcome measures such as time spent on each case. The participants were asked to self-report on the fidelity of simulation, user experience metrics, workflow metrics, clinical consultations, patient concerns, and care decisions. This was done using postcase surveys, postcase conduct surveys, postsimulation surveys, or a postsimulation interview. Each of these was measured using various questions.

**Table 2.** Methods used to report on the study outcomes.

Measure	Moderator measured	Participant neurologist reported			
		Survey after case preparation	Survey after case conduct	Survey after simulation	Interview after simulation
Fidelity of simulation					
Similarity of patient cases to those seen in day-to-day practice				**X**	
Similarity of simulation to day-to-day practice consultations				**X**	
Using clinical simulation to evaluate the Floodlight MS^a^ HCP^b^ Portal				**X**	**X**
User experience metrics					
User feedback on the Floodlight MS HCP Portal				**X**	**X**
Usability of the Floodlight MS HCP Portal				**X**	
Workflow metrics					
Time taken to prepare for teleconsultation	**X**				
Time taken to conduct teleconsultation	**X**				
Reported mental effort associated with information acquisition and application		**X**	**X**		
Clinical consultations, patient concerns, and care decisions					
Reported confidence level when preparing for and making care decisions during a teleconsultation		**X**	**X**		
Identification of the patient’s primary concern		**X**	**X**		**X**
Time taken to identify the patient’s primary concern during teleconsultation (independently verified by a second clinician using screen recordings)	**X**				
Recognition of “invisible symptoms of MS”^c^ and their proactive discussion with patients	**X**		**X**		
Care decisions taken and reported prompts for these decisions	**X**		**X**		**X**

a MS: multiple sclerosis.

b HCP: health care professional.

c Invisible symptoms, including fatigue, mood disorders, cognitive impairments, pain, bladder or bowel dysfunction, sexual dysfunction, and vision changes can be experienced by people living with MS. Some of these symptoms may not be externally evident or easy to report and quantify and can be difficult for the patient to report and articulate during a clinical encounter (eg, due to subjectivity bias, poor recall, or stigma) or for physicians to capture (eg, limitations in available measurement tools, ample reliance on patient recall on the visit day).

### Data Analysis

For continuous, time-dependent, moderator-assessed measures, the median times were calculated and presented as box and whisker diagrams. Additionally, these time-dependent measures were collated into groups, and all categorical results were presented as absolute numbers within each category and presented as bar charts. An a priori decision was made not to perform hypothesis-testing statistics on data from this feasibility study.

### Ethical Considerations

CONSONANCE patient data anonymity was retained throughout the preparation and conduct of this study. The protocol (22-PROH-102: Evaluation of Floodlight MS using clinical simulation) was reviewed by Pearl Institutional Review Board on September 20, 2022, and determined to be exempt from ethics approval according to U.S. Food and Drug Administration 21 Code of Federal Regulations (CFR) 56.104 and 45CFR46.104(b)(3): (3) Benign Behavioral Interventions - Adults. The study was conducted in accordance with the most recent version of the Declaration of Helsinki. Signed consent forms were obtained from the participant neurologists before the simulated teleconsultations were conducted. Participant neurologists were compensated for their time according to local Fair Market Value rates.

## Results

### Participant Neurologist Demographics

A total of 10 US-based neurologists were recruited as participants (7 from large academic centers and 3 from specialist neurology centers). A total of 6 participant neurologists had >10 years of experience in performing MS consultations, 1 had 5‐10 years of experience, and 3 had 3‐5 years of experience. The number of consultations performed by participant neurologists in their current practice in a typical week was >50 (n=2), 40‐50 (n=1), 30‐40 (n=1), 20‐30 (n=3), or 10‐20 (n=3). Further, participant neurologists performed 25%‐50% (n=3) or <25% (n=7) of all consultations as teleconsultations in their current practice.

### Patient Case and Simulation Fidelity

Participant neurologists felt that the simulations were high-fidelity representations of real consultations. All reported that the patient cases and scenarios were “very” or “somewhat” similar to typical practice (Table 3). Feedback from participants on the performance of the professional patient actor was mostly very positive:

Shockingly. He was very good. I say that. I haven’t done this in a while, but very often a mock patient doesn’t get the real essence of MS. This guy did great. I even wondered, did he have MS, how he got so good at it? ... They would have had to practice and discuss things with many patients and doctors to get this good.

The actor did a great job. Intuitively knew how to pick up on what I was asking.

**Table 3.** Participant neurologists’ experiences of patient case and simulation fidelity.^a^

Participant neurologists’ experiences	Number of participant neurologists				
	Very different	Somewhat different	Neutral	Somewhat similar	Very similar
How similar were these patient cases to those you see in your day-to-day practice?	0	0	0	4	6
How similar were these consultations to the consultations you complete during a typical week?	0	0	0	5	5

a Participant neurologists reported similarity of patient cases to those seen in day-to-day practice and similarity of simulation to day-to-day practice consultations via surveys and questionnaires.

### User Experience Metrics

**Figure 3.**
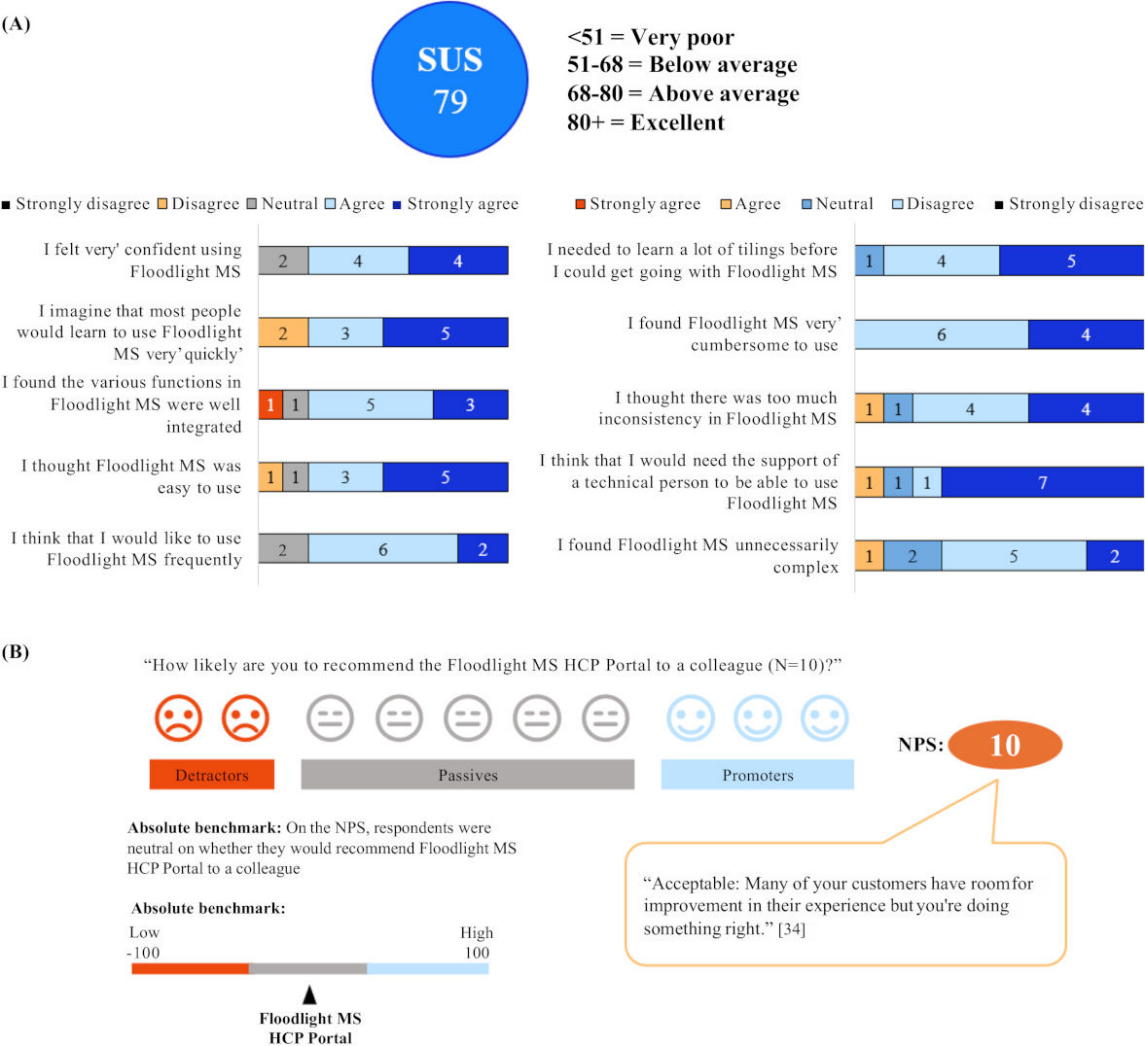
User experience metrics for the Floodlight technology, including (A) SUS and (B) NPS [34]. The bars represent responses on the user experience of Floodlight MS (SUS) and user experience for data visualization and the overall experience (NPS); these were reported via participant surveys and interviews. Items alternate between positive and negative phrasing, with higher scores indicating more favorable usability perceptions. HCP: health care professional; MS: multiple sclerosis; NPS: net promoter score; SUS: System Usability Scale.

The Floodlight MS HCP Portal scored an “above average” score of 79 on the System Usability Scale (Figure 3A). In terms of overall experience of using the Floodlight technology, the Floodlight MS HCP Portal scored an “acceptable” Net Promoter Score of 10, based on the participant neurologists’ reports of how likely they would be to recommend it to a colleague (Figure 3B).

**Figure 4.**
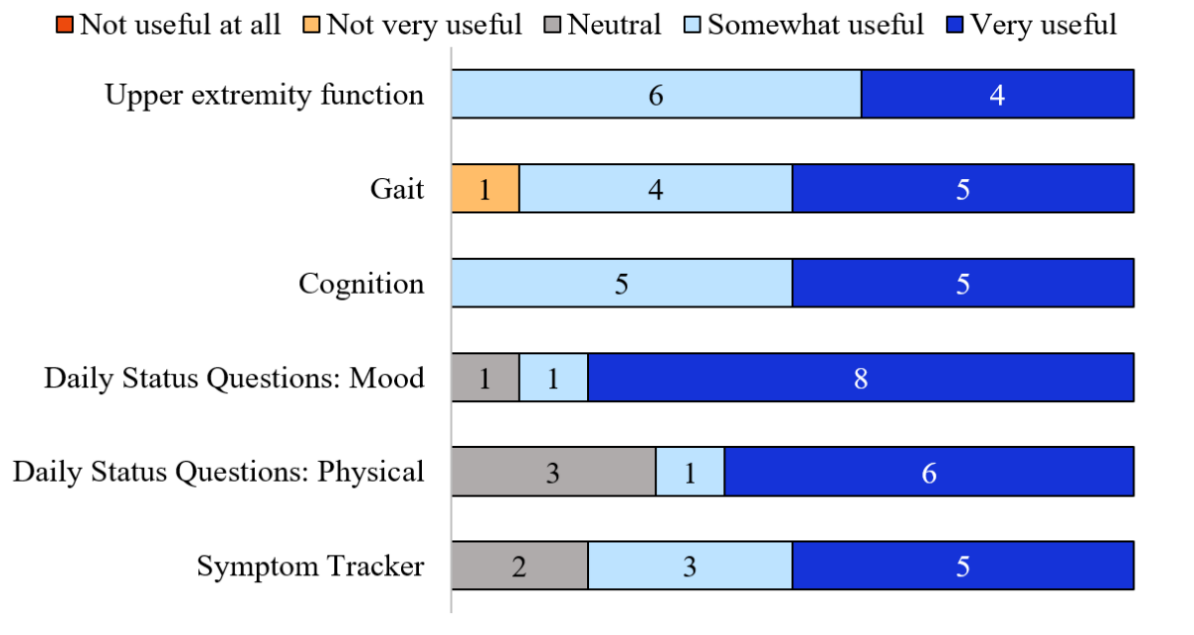
User experience metrics for the usefulness of data visualizations in the Floodlight MS HCP Portal. Participant neurologists provided feedback on whether data visualizations in the Floodlight MS HCP Portal were useful for factors including upper extremity function, gait, and cognition. HCP: health care professional; MS: multiple sclerosis.

Participant neurologists agreed that data visualizations in the Floodlight MS HCP Portal were “very” or “somewhat” useful for upper extremity function (10/10), cognition (10/10), gait (9/10), and mood status (obtained via the Floodlight Daily Status Questions; 9/10; Figure 4). The “mood status” aspect of the Patient Journal was rated as the most useful of the data visualizations presented, with 8/10 ratings of “very useful”.

### Workflow Metrics

**Figure 5.**
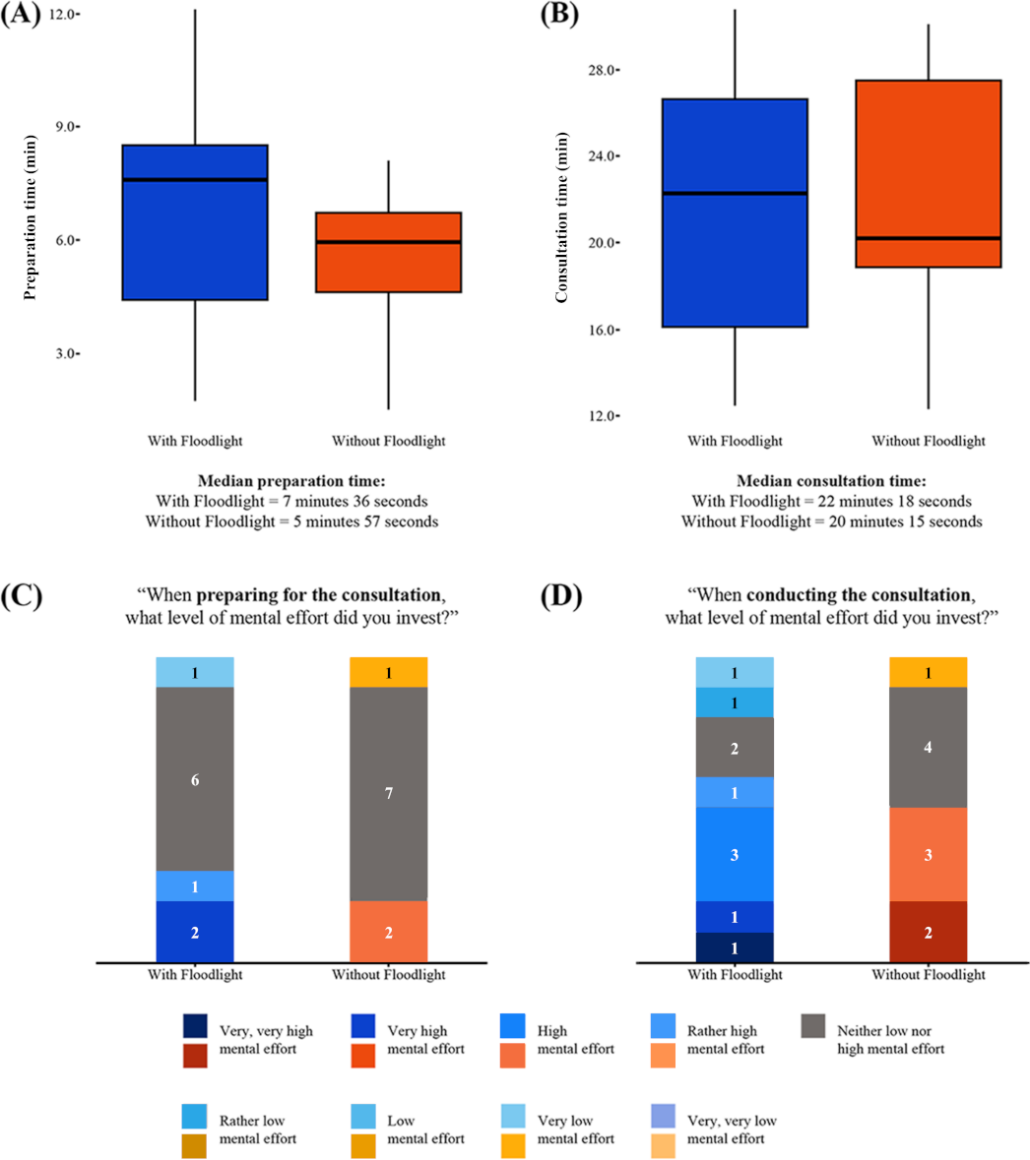
Workflow metrics. (A) Preparation time for patient cases with and without Floodlight data. (B) Teleconsultation time with and without Floodlight data. (C) Mental effort required preparing for patient cases with and without Floodlight data. (D) Mental effort required when conducting patient cases with and without Floodlight data. The classification key uses shades of blue for data obtained “with Floodlight” and shades of red for data obtained “without Floodlight”; shade of gray uses the same classification in both “with Floodlight” and “without Floodlight”.

In terms of the time taken for patient case preparation, cases took a median of 7 minutes and 36 seconds with Floodlight and 5 minutes and 57 seconds without Floodlight (Figure 5A). The median time taken to conduct the teleconsultations was 22 minutes with Floodlight and 20 minutes without Floodlight (Figure 5B). When using the Floodlight technology, participant neurologists also reported investing a slightly higher level of mental effort to prepare for (Figure 5C; very high mental effort to rather high mental effort: n=3 vs n=2) and conduct patient cases (Figure 5D; very, very high mental effort to rather high mental effort: n=6 vs n=5).

### Clinical Consultations and Patient Concerns

**Figure 6.**
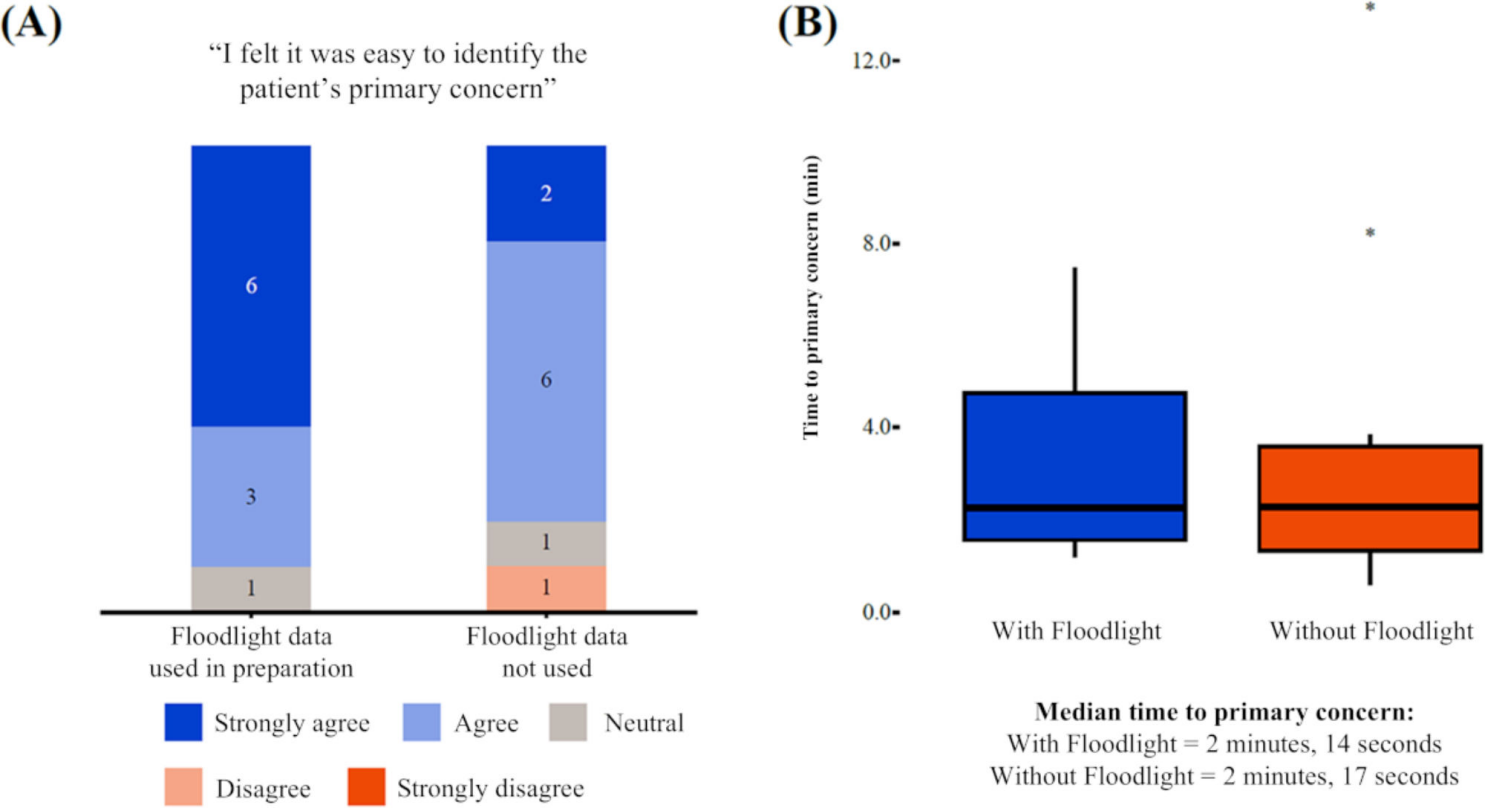
Patients’ primary domains of concern: (A) identification and (B) time taken. The identification of patient concern and the median time taken for participant neurologists to initiate discussions on the primary domain of concern were assessed during the consultation preparation phase with and without access to Floodlight data.

About 6 out of the 10 neurologists “strongly agreed” that it was easier to identify patient concerns within the relevant functional domains with access to Floodlight data during the consultation preparation phase versus without access (where 2/10 “strongly agreed”; Figure 6A). The median time taken for participant neurologists to start discussing the primary domain of concern was 2 minutes, 14 seconds when using the Floodlight technology and 2 minutes, and 17 seconds without the Floodlight technology (Figure 6B).

### Recognition of “Invisible” Symptoms of MS

The median time taken to discuss each “invisible” symptom was between 1 and 1.5 minutes (Table 4). In Patient Case A, which was structured to show a prominent cognitive decline, cognitive issues, and mood change, discussions exceeded 2 minutes. In Patient Case B, bladder dysfunction (which was referred to in the clinical letters and without the support of Floodlight information) was also discussed for over 2 minutes. While differences can be gleaned at the individual symptom level, there was no clear pattern to the length of the discussions. Being aware of the symptom by means of the clinical letters did not necessarily seem to be a clear determinant in the participant neurologist spending more time on discussions with the patient actor. There were also no consistent differences in the median time spent discussing symptoms across domains with and without the use of the Floodlight technology.

**Table 4.** Median time spent discussing each “invisible” symptom^a^ with patients (such as fatigue and bladder dysfunction that are difficult to evaluate by health care professionals [HCPs]) across all teleconsultations with and without the Floodlight technology.

Symptom	Patient Case A^b^		Patient Case B^b^	
	With Floodlight (min)	Without Floodlight (min)	With Floodlight (min)	Without Floodlight (min)
Vision changes	01:32*^b^*	01:18^b^	00:30	00:10
Sexual dysfunction	00:05	01:15	00:17^b^	00:55^b^
Mood changes	02:18	01:26	01:32^b^	01:50^b^
Physical and emotional pain	01:55	01:15	*0*1:54^b^	01:12^b^
Bowel and bladder dysfunction	00:30^b^	01:10^b^	01:14^b^	02:47^b^
Cognitive changes	02:16^b^	02:06^b^	00:27	00:53
Fatigue	00:40^b^	01:01^b^	01:08	01:39
Median across all domains	01:25	01:22	01:07	01:34

a Invisible symptoms, including fatigue, mood disorders, cognitive impairments, pain, bladder or bowel dysfunction, sexual dysfunction, and vision changes can be experienced by people living with MS. Some of these symptoms may not be externally evident or easy to report and quantify and can be difficult for the patient to report and articulate during a clinical encounter (eg, due to subjectivity bias, poor recall, or stigma) or for physicians to capture (eg, limitations in available measurement tools, ample reliance on patient recall on the visit day).

b These are the symptoms that were previously reported, per the clinical documentation provided, for Patient Case A and Patient Case B.

### Care Decisions and Change in Disease Management Decisions

**Figure 7.**
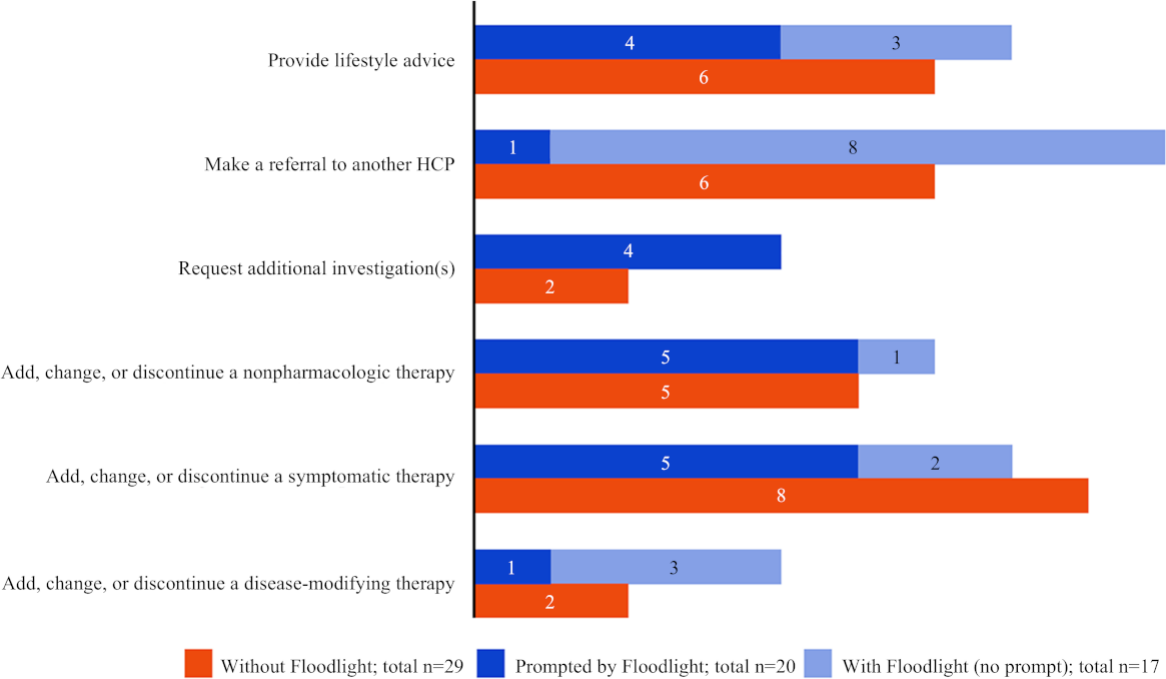
Care decisions, including change in disease management, prompted by Floodlight data and taken when using the Floodlight technology, versus without. Floodlight data were reported as a trigger for care and change in disease management decisions, including regarding disease-modifying therapies, additional medications, referrals, or investigations. In all instances, Floodlight data were used in conjunction with other triggers such as patient history or clinical exam findings. HCP: health care professional.

Floodlight data were reported as a trigger for 20 of the 37 care and change in disease management decisions (including regarding disease-modifying therapies, additional medications, referrals, or investigations), always in combination with other triggers such as patient history (20/20) or clinical exam findings (9/20; Figure 7). Overall, a greater number of care decisions were taken when using the Floodlight technology (37) versus without (29), including making a referral to another HCP (9 vs 6), or adding, changing, or discontinuing a nonpharmacologic therapy (6 vs 5) or disease-modifying therapy (4 vs 2).

### Overall Feedback

Overall, participant neurologists valued access to longitudinal data, particularly regarding hand function and cognition. Many participant neurologists found the aspects of the user interface challenging when using the Floodlight MS HCP Portal. They felt that user interface improvements were the most important changes to be made, and they noted the following could help improve the interface: “Separating different data points and putting them into separate charts,” “...an overall curve, visualizing the trend over time,” and “…being able to change the y axis to separate data points for easier review.”

### Data Availability

For up-to-date details on Roche’s global policy on the sharing of clinical information and how to request access to related clinical study documents, please visit [35].

## Discussion

### Principal Findings

The clinical simulations of MS teleconsultations described in this study demonstrate the ability of this research methodology to deliver high-fidelity patient cases. They also enabled direct comparison of teleconsultations conducted with and without the use of digital health technologies.

Clinical simulation offered several advantages, including the ability to present relevant, representative, paradigmatic MS cases and to efficiently generate insights on the utility and usability of new digital technology in clinical visits. This provided early real-world insights from neurologists who were naïve to the Floodlight technology and who received only brief introductory training on its use. Specifically, it provided an opportunity to move beyond the expected impact of the tool and, instead, explore the approach used by participants naïve to the digital technology (after a brief orientation) in actual clinical decision-making.

Using real patient data from the CONSONANCE study enabled access to extensive existing data without additional patient consent or data acquisition, allowing case selection based on in-depth clinical information. The richness of the CONSONANCE dataset minimized the need for synthetic data, which was limited but where used, derived from valid sources such as narrative radiology reports and clinical letters. While this approach ensured high-fidelity and clinically relevant patient scenarios, it introduced a limitation in the generalizability of the simulated cases as the patient population in the CONSONANCE study was composed entirely of people with progressive multiple sclerosis. This highlights the capability of clinical simulation to leverage rich, real-world datasets while also underscoring the importance of including a broader spectrum of patient profiles and validated synthetic cases to enhance external validity, scalability, and applicability across diverse MS phenotypes. Additional simulated patient cases can be easily generated from other clinical trials or by generating synthetic (but realistic) patient case data. Further simulation research should expand the number and diversity of patient cases, including data from varied trial populations and validated synthetic cases to better capture the heterogeneity of MS care. It is important to ensure that these cases are representative of typical patients seen in clinical practice and developed with appropriate ethical considerations to avoid the introduction of unwanted bias. Additionally, it is also essential to ensure that clinical trial data used for patient cases do not allow patient identities to be inferred and that blinding procedures are preserved.

This study explored complex MS cases, with multiple patient concerns and functional domains affected, to capture the complexity of real-world MS clinical visits and explore the impact of utilizing a digital tool (Floodlight technology). As with “real” clinical encounters with people living with MS, distilling prior history and functional testing into condensed clinical letters for use in the consultations was challenging, due to the heterogeneity of symptoms, scales, and other assessments used. For example, bladder symptoms reported by patients in the SymptoMScreen were consistently underscored in the clinicians’ EDSS Bowel/Bladder Functional System [36-38].

Research applications of simulation can include training methodologies for educational purposes [22], such as in training for medical students or less experienced HCPs, and to enhance clinical competence [21]. Additionally, simulation can be used to improve primary care office–based readiness to respond to medical emergencies [39] or to enhance cultural sensitivity, communication, and empathy among HCPs [40]. Simulation can also be used as an investigative methodology to answer research questions that otherwise could not be answered feasibly, safely, ethically, or in a timely fashion in clinical settings [22], as pertains to our study.

The simulation methodology provided key insights into integrating a digital health tool into routine MS care. Initial findings pertained to how neurologists naïve to this technology began to access and interpret these data. Indeed, there is a known socialization process required for the adoption of new technology [41], and this must be accounted for in the plans that developers have for the implementation of new tools. Neurologists scored the clinician-facing HCP Portal for accessing Floodlight MS data “above average” on the System Usability Scale, indicating a high level of usability. Feedback from postsimulation surveys and interviews provides a foundation for iterative improvements to the user interface. Among the available data on the portal, the “mood status” aspect of the Patient Journal (Daily Status Questions) was rated as the most useful of the data visualizations presented, which is potentially indicative of a medical need (eg, more systematic depression monitoring). Finally, as expected, incorporating Floodlight data into teleconsultation preparation required slightly more time and mental effort than consultation without such technology. Interestingly, the median time was only 1.7 minutes longer for patient case preparation, which seems relatively modest compared with the potential gains from having Floodlight data available. This also indicates that the brief presimulation training was effective and supports the tool’s scalability. With increased familiarity, time required to use the Floodlight technology during the visits would likely decrease. However, even a short amount of additional time requirement could nonetheless present a potential barrier to use. For example, when evaluating the initial launch of the patient codesigned, high-usability-scoring, point-of-care clinical dashboard MS NeuroShare, clinicians were less likely to use it on busy clinic days, despite the reported improvements in the quality of communication and patient understanding of management care plans. New solutions to alleviate clinician workloads must be evaluated against the time required to implement them, such as educating patients on the clinical applications [41].

Digital health tools can capture objective data between clinical visits. This can avoid reliance on patient recall of symptoms, helping to provide clinicians with a more complete picture of symptoms and enabling discussions between HCPs and patients. The second set of exploratory insights, therefore, concerned the impact that ready access to Floodlight data might have on clinical discussion and management. Overall, while participant neurologists started discussing the primary domain of concern after 2 minutes both with or without the Floodlight technology, most participants said that it was easier to identify patients’ concerns when they had access to the Floodlight MS HCP Portal (Figure 6A). Further, more care decisions were taken when using Floodlight data versus without, for example, making a referral to another HCP or amending a disease-modifying therapy.

### Limitations

While simulations can represent and provide insights on the dynamics of MS in real-life clinical practice, the method is limited by the ability to account for operational and behavioral variables that occur in clinical practices, and it may, therefore, not be a fully accurate representation. While this methodology is inherently scalable, its primary limitation lies in the effort needed to recruit and train actors. By comparison, on-site studies involving real patients are even more constrained, as they can only include individuals residing near the clinical study site(s). Although the actors performed convincingly, simulations cannot fully substitute for real patient interactions. Real patients may experience factors such as anxiety about their clinical outcomes, fatigue from poor sleep, or depression, all of which can influence the dynamics of clinical encounters; however, it must be noted that this is not one of the aims of this study. The limited numbers of participants and patient cases in this study, important for this initial testing of the research methodology, naturally limit generalizability to real-life clinical consultations. Virtual simulations could enrich the recruitment of diverse neurologists by offering more convenient participation as well as the potential to recruit neurologists from a wider geographic area. This will be important in studies that seek to quantify differences in clinical decision-making. Furthermore, a potential source of bias relevant to all simulations involving observation of tasks is the Hawthorne effect, where participants modify their behavior when they know they are being observed [42,43]. Mitigation strategies to overcome this included informing participating neurologists in advance about the study objectives, emphasizing that the research focus was not primarily on diagnostic accuracy, and assuring them that their identities would remain confidential. To further minimize the Hawthorne effect, the observer’s camera and microphone were switched off during sessions; however, the observer remained present on the call to ensure study fidelity and provide technical support if needed. It should be acknowledged that despite these measures, such biases cannot be entirely eliminated in simulation-based research, although increasing the number of participants might reduce their impact.

Further, exploring cognitive impairment as a primary domain of concern highlighted some limitations of using simulated encounters because some participant neurologists described typically engaging patients’ family members or care partners when assessing the extent and impact of a patient’s cognitive challenges, and family members were not included in the simulated scenarios. An alternative approach in clinical simulations exploring more focused digital tools would be to curate simpler patient cases with only 1 domain of concern and a few specific invisible symptoms and to focus the investigation on more specific aspects.

### Conclusions

Clinical simulation of MS teleconsultations allowed evidence generation on the potential utility of a digital health technology tool in MS care. The simulated patient cases and environment were found to be of high fidelity and enabled the direct comparison of teleconsultations conducted with and without the use of the Floodlight technology. A number of domains could be explored, including tool usability, influence of Floodlight data on clinical decisions, and impact on clinical workflows. Employing this methodology provided preliminary evidence that the Floodlight technology could support routine MS care, as it was found to be usable and useful, with minimal additional time and mental effort required on the part of neurologists previously naïve to the technology.

The findings of this study support expanded research into and development of clinical simulation methodologies for neurological research. This could expedite integrating innovative methods or interventions in health care settings, as well as in medical training. In the digital health space specifically, simulations could help to anticipate what socialization of technologies may require, to steer guidance on usage, explain their value to clinicians, and help health systems plan implementation.

## Abbreviations

EDSS: Expanded Disability Status Scale
HCP: health care professional
MS: multiple sclerosis
PRO: patient-reported outcome
SDMT: Symbol Digit Modalities Test
